# A pipeline for making ^31^P NMR accessible for small- and large-scale lipidomics studies

**DOI:** 10.1007/s00216-021-03430-4

**Published:** 2021-07-12

**Authors:** Samuel Furse, Huw E. L. Williams, Adam J. Watkins, Samuel Virtue, Antonio Vidal-Puig, Risha Amarsi, Marika Charalambous, Albert Koulman

**Affiliations:** 1 Core Metabolomics and Lipidomics Laboratory, Wellcome Trust-MRC Institute of Metabolic Science-Metabolic Research Laboratories, University of Cambridge, Cambridge, CB2 0QQ UK; 2 Metabolic Disease Unit, Wellcome Trust-MRC Institute of Metabolic Science-Metabolic Research Laboratories, University of Cambridge, Cambridge, CB2 0QQ UK; 3 Biological Chemistry Group, Jodrell Laboratory, Royal Botanic Gardens Kew, Richmond, TW9 3AE UK; 4 Biodiscovery Institute, University of Nottingham, University Park, Nottingham, NG7 2RD UK; 5 Division of Child Health, Obstetrics and Gynaecology, Faculty of Medicine, University of Nottingham, Nottingham, NG7 2UH UK; 6 Department of Medical and Molecular Genetics, King’s College London, Guys Hospital, WC2R 2LS, London, UK

**Keywords:** Lipidomics, Lipid profiling, ^31^P NMR

## Abstract

Detailed molecular analysis is of increasing importance in research into the regulation of biochemical pathways, organismal growth and disease. Lipidomics in particular is increasingly sought after as it provides insight into molecular species involved in energy storage, signalling and fundamental cellular structures. This has led to the use of a range of tools and techniques to acquire lipidomics data. ^31^P NMR for lipidomics offers well-resolved head group/lipid class analysis, structural data that can be used to inform and strengthen interpretation of mass spectrometry data and part of a priori structural determination. In the present study, we codify the use of ^31^P NMR for lipidomics studies to make the technique more accessible to new users and more useful for a wider range of questions. The technique can be used in isolation (phospholipidomics) or as a part of determining lipid composition (lipidomics). We describe the process from sample extraction to data processing and analysis. This pipeline is important because it allows greater thoroughness in lipidomics studies and increases scope for answering scientific questions about lipid-containing systems.

**Supplementary Information:**

The online version contains supplementary material available at 10.1007/s00216-021-03430-4.

## Introduction

Lipidomics is of increasing importance in studies of living systems as phospholipids, triglycerides and sterols have various roles in metabolic disease, growth, infection and the structure of biological systems [1–12]. Lipidomics is complementary to approaches such as proteomics and transcriptomics, and relatively quick to acquire and therefore cost effective. Furthermore, an increasing array of techniques is becoming available that make storing and extracting lipids from fibrous and other sample types more efficient and consistent [13–15]. This makes lipidomics an increasingly attractive part of hypothesis-driven research in biology.

The best strategy for acquiring lipidomics data from a large number of samples in a single analytical batch is to use high-throughput approaches such as high resolution mass spectrometry, typically Direct Infusion (DIMS) [16, 17]. This enables large numbers of variables to be quantified in a large number of samples relatively quickly, meaning the sample numbers required for RCTs can be profiled in a single analytical batch. However, the lack of chromatography and the reliance on *m/z* only for identification means that isobaric species are indistinguishable. This problem can be partially solved by hyphenating liquid chromatography with MS (LCMS), which can separate analytes chromatographically before detection. Still, all MS based methodologies rely on the ionization efficiency of lipids and although internal standards can be used to correct for this, many aspects that influence ionization efficiency and ion suppression are not comprehensively understood. Therefore, neither DIMS nor LCMS is capable of offering unambiguous structural data, meaning that MS can lack specificity and clarity.

An orthogonal method of data acquisition can help to overcome these fundamental limitations. An established technique that is orthogonal to MS and useful for determining molecular structure precisely is NMR. NMR is based on the molecular environment of atomic nuclei with a spin of ½, with most open-access spectrometers at Universities running experiments on one or more of ^1^H, ^13^C, ^31^P and ^19^F. ^31^P NMR in particular is useful in lipidomics as it can be used for all phospholipids. Part of the reason for this is that all molecules that do not contain phosphorus are in effect invisible. A tailor-made solvent mixture that maximizes solubility of phospholipids and gives good separation of resonances whilst limiting that of polar and non-polar material (such as sugars and triglycerides, respectively), has been developed for this purpose [18–21]. The availability of such a solvent system has led to ^31^P NMR being used as the sole technique for acquiring lipidomics data for some low- and medium-throughput studies [22–25].

^31^P NMR is useful for providing a straightforward spectrum of lipid class abundance but can also be used to acquire data about the molecular structure of lipids and allows us to correct for different ionization efficiencies in in MS lipidomics data. These principles are the foundation of the combination of NMR and MS for lipidomics known as dual spectroscopy [13]. It can also be used to acquire all the lipidomics data required for studies in which only molar concentration is required, e.g*.* structural studies [25]. However, many of the lipidomics-specific aspects of ^31^P NMR have not yet been described in detail and the technique is typically alien to users not already familiar with NMR. This has made the employment of ^31^P NMR in routine lipidomics difficult or impossible for most labs.

**Fig. 1:**
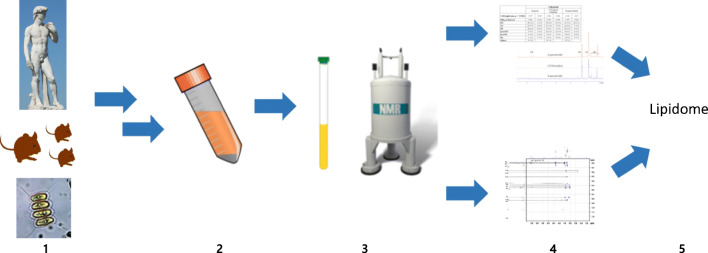
The sequence of steps required in acquiring a lipidome. Tissues from biological samples appropriate to the study (1) are isolated and homogenized as appropriate for extraction (2) and sample prepared and run (3). Data acquisition and processing (4) yields the lipidome (5). The present study codifies the requirements of steps going from sample preparation to data analysis as a pipeline

This barrier to the use of ^31^P NMR as a technique for lipidomics led us to develop a dedicated pipeline for it. We have tested the limits of this technique and designed an approach for practical application of this method either independently or as part of a larger lipidomics programme. The purpose of this is to facilitate lipidomics across a range of fields in biology in which lipidomics is being used. The outline of this pipeline is shown in Fig. 1.

The limitations of the technique and the tools developed were tested using a variety of samples. Lecithin from *Glycine max* was used to establish the relationship between sample size/mass and signal strength—valuable for determining minimum sample volumes and/or numbers of acquisition scans. A variety of mouse tissues were also used to identify a full range of phospholipids and their shifts. Lecithin was also used to explore the relationship between sample age and lipid profile, several resonances (through adducts) and stretching.

This pipeline is important as it facilitates the use of another technique for lipidomics and increases that technique’s applicability. It also adds to the increasing use of phosphorus^31^P NMR in metabolomics [26]. The increasing desire and reliance upon lipidomics and metabolomics data for answering biological questions creates a demand for a choice of finely-drawn methods, not only for a straightforward answer as to the composition of a system but also approaches for verification and validation of data to increase confidence in the omics data acquired. The present pipeline is designed to be flexible and offer approaches to all these needs.

## Methods

### Materials, consumables and chemicals

Solvents, reagents, fine chemicals and NMR tubes were purchased from SigmaAldrich (Gillingham, Dorset, UK). Lecithin lipid mixture was purchased from Holland and Barrett in 2020 and not purified further.

### Animal model

All biological samples were isolated from experimental mice used under ethically-approved studies. All procedures were conducted in accordance with the UK Home Office Animal (Scientific Procedures) Act 1986 and local ethics committees at either Aston University, King’s College London and the Universities of Nottingham and Cambridge, as described previously [17, 27–29].

### Solvent system for solution phase NMR

Samples were dissolved in freshly-made stock solutions of a modified [13, 22] form of the ‘CUBO’ solvent system [18–21] in which the amount of dueteriated dimethylformamide *d*_7_-DMF was minimized. Stocks comprised dimethylformamide (3·5 mL), *d*_7_-DMF (1·5 mL), triethylamine (1·5 mL) and guanidinium chloride (500 mg) or multiples thereof.

### Tissue sample preparation

Fibrous animal tissue samples were prepared and extracted as described recently [13, 30]. Briefly, the tissue of interest was homogenized with a hand-held homogenizer in GCTU to give an homogenous, pipettable solution with at least 1 μL lipid/60 μL homogenate that was stored at −80 °C until extraction.

### Lipid extraction and sample preparation for ^31^P NMR

Appropriate tissue homogenates were combined to give 5–10 mg of phospholipid per NMR sample. The extraction was as described previously [13, 15]. Briefly, pooled homogenates (5–8 mL) were diluted (stock solution of DMT, 50 mL, Falcon tube) and agitated before centrifugation (3·2 k × *g*, 2 min). The aqueous fraction and the mesophasic solid were discarded, and the organic solution dried under a flow of nitrogen. The resulting lipid films were dispersed in CUBO immediately (700 μL) and transferred (~650 μL) to a Wilmad® 507PP tube and queued for data acquisition within 72 h.

### NMR spectrometer and probe (1D).

 Lipid samples were run on a Bruker Avance Neo 800 MHz spectrometer, equipped with a QCI cryoprobe probe. 1D Phosphorous experiments were acquired using inverse gated proton decoupling. Spectra were averaged over 1312 transients with 3882 complex points with a spectral width of 14·98 ppm. An overall recovery delay of 8·4 s was used. Data were processed using an exponential line broadening window function of 1.5 Hz prior to zero filling to 32,768 points and Fourier transform. Subsequent integrations above a noise threshold of 0·01% of the total ^31^P were used to establish a given phosphorus environment’s molar quantity.

### NMR spectrometer and probe (2D).

^31^P-HSQC spectra were acquired using an XY16-CPMG sequence for transfer and GARP-4 decoupling. The direct dimension consisted of 3072 points with 16 transients, and 2048 points in *t*1 collected using non-uniform sampling with a 25% sparsity, giving 512 points overall for the indirect dimension. Data were processed with compressed sensing, linear prediction in F1 and a shifted sine squared window functions.

### Data processing and statistical methods.

 NMR data were processed and deconvoluted using TopSpin 4.0.7. Signal-to-noise ratios were calculated using NMR*Proc*Flow 1.3 online. Univariate and bivariate statistical calculations were made using Microsoft Excel 2016. Graphs were prepared in Excel 2016.

## Results & discussion

A pipeline for describing the use of ^31^P NMR in lipidomics requires a detailed investigation of several steps (Fig. 1). However some of these may not be obvious. Before data are acquired, the sample must be dissolved, remain stable, be concentrated enough and the pulse sequence used by the instrument must be appropriate for the hypothesis being tested (e.g. suitable for quantitative measurements). Once data are collected, identification of resonances by shift and couplings is required. All the relevant steps are described in order below.

### Solvent system

The desire to acquire lipidomics data using ^31^P NMR has led to the development of solution phase solvent systems for the purpose. Several solvent systems have been tested, including detergent-based systems [31–33] and chloroform-methanol mixtures [34–37]. However, high viscosity and difficulty in recovering the sample (detergent systems), and relatively low solubility of lipids (chlorinated solvents) have limited the use of these particular solvent systems in detailed lipidomics studies involving batches of samples. Furthermore, both of these solvent systems can dissolve phosphorus-containing compounds that are not lipids, adding to the number of signals observed thus adding noise.

These limits led to the development of a solvent system tailored for ^31^P NMR lipidomics, originally described as the ‘CUBO’ mixture [19]. It is more effective than other systems due to its ability to dissolve larger sample masses, specificity for phosphorus-containing lipids, with improved resolution and lower viscosity. The sample can also be recovered with relative ease. Several tests of CUBO against chloroform-methanol mixtures have confirmed this [18–20], with comparisons between spectra from samples with detergent-based solvent systems and CUBO showing better resolution in the latter. The CUBO system has been developed further by us to include a source of deuterium (*d*_7_*-*DMF) that allows such samples to be locked during preparation for data acquisition, and thus be run more easily [38]. Around 20% *d*_7_*-*DMF is typically enough for locking in experiments on open-access instruments. The modified CUBO solvent system has a remarkable tolerance to the amount of lipid in the sample, around 1·5 orders of magnitude from the minimum detectable. Samples can be prepared simply (see *Methods*) by dissolving lipid films in the solvent and decanting to the glass tubes.

### Sample concentration

**Fig. 2:**
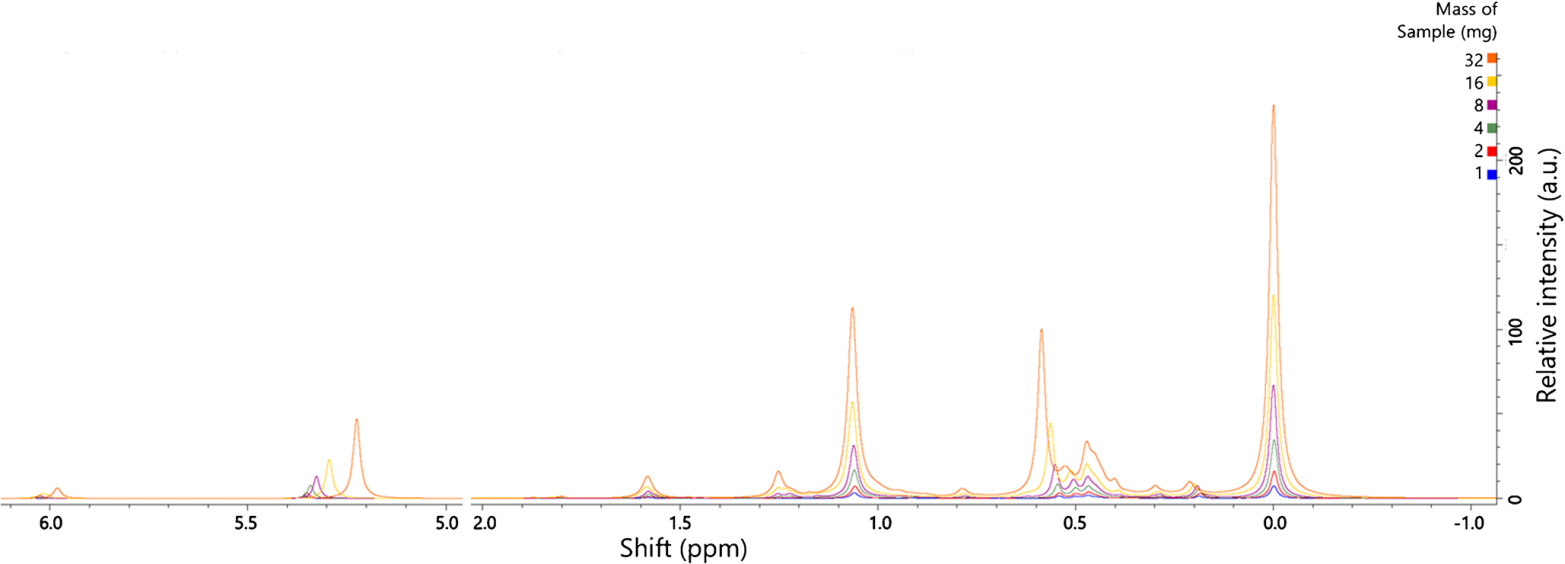
Sample concentration scan. Samples comprised Lecithin extracts from *Glycine max*, run 48-72 h after sample preparation. Samples prepared in the modified CUBO solvent system, 650 μL/sample (13, 22), run at 293 K. *y* axis shows relative intensity (arbitrary units), *x* axis shows chemical shift (ppm). σ_P_ (324 MHz, CUBO, ppm) 6·1–5·9, *lyso*-phosphatidic acid; 5·4–5·2, phosphatidic acid; 1·65–1·55, *lyso*-phosphatidylinositol; 1·28–1·20, phosphatidylglycerol; 1·15–0·95, phosphatidylglycerol; 0·85–0·80, sphingomyelin; 0·60–0·57, phosphatidylethanolamine; 0·57–0·55, phosphatidylethanolamine; 0·55–0·51, phosphatidylethanolamine; 0·50–0·48, phosphatidylethanolamine; 0·46–0·43, *lyso*-phosphatidylcholine; 0·30–0·25, unk.; 0·19–0·16 unk.; 0·00, phosphatidylcholine (calibration reference)

The amount of sample available is often limited in metabolomics studies, especially in human trials. It is therefore essential to know the minimum mass of the mixture of phospholipids required to produce a viable ^31^P NMR sample and how samples of different concentrations compare. The concentration of analytes in a given biological sample may be quite different to others and so assessment of the phospholipid concentration in a sample type of interest is useful before collection of the samples for a study. We tested the quality of spectra against the mass of lipid per sample. Phospholipid (lecithin) extracted from *Glycine max* was used to explore this as it was a lipid mixture available in quantity that comprised a variety of commonplace lipids. A concentration scan of lecithin (1·0–32·0 mg/650 μL, Fig. 2*,* data acquired 48 h after sample preparation) showed that the shift of some resonances is concentration-dependent with respect to phosphatidylcholine (referenced to 0·00 ppm, with other signals assigned as per previous reports [13, 18–22, 38]).

Phosphatidylethanolamine (PE, 0·60–0·50 ppm) shifted downfield with increasing concentration, where phosphatidic acid (PA, 5·32–5·25 ppm) and *lyso-*phosphatidic acid (LPA, 6·03–5·96 ppm) moved upfield through the scan. The more upfield of the two signals associated with phosphatidylglycerol (PG, 1·24–1·21 ppm) moved downfield with increasing concentration, appearing as one broad resonance in the 32 mg sample. However, the shifts assigned as *lyso-*phosphatidylcholine and phosphatidylinositol (LPC, PI) were not concentration-dependent in this range.

This relationship between concentration and shift raised questions about what threshold should be used for regarding a lipid as ‘present’. Generally, in metabolomics and lipidomics, a signal to noise (S/N) ratio of 3:1 is required for a signal to be regarded as genuine. We therefore plotted the S/N for resonances of samples in this concentration span to characterize the relationship between concentration and signal strength (see Supplementary Information (ESM) Fig. S1). The width of the concentration scan of lecithin showed that the minimum mass of phospholipid concentrate for an S/N of 3 is 1 mg/sample for resonances PA, PC, PE, PI and PS (ESM Fig. S1*,* log(3) = 0·477). Samples of higher concentration provide better resolution and of yet more signals, with LPA, CL and PG showing a S/N of >3 from a sample size of 4 mg. However, the S/N does not continue linearly above 16 mg for one of the resonances assigned to PE. This suggests that the concentration range of 4–16 mg is generally the optimum range for mixed lipid samples in an 800 MHz field with 656 scans. We therefore support the suggestion for pooling samples for each phenotype in human trials [13] to compare these groups using ^31^P NMR. These results suggest that a minimum of 4 mg of lipids from biological extracts is required for a reliable ^31^P NMR sample of typical mixed lipids. Although this may be challenging for some human studies, this sample size is more achievable, even desirable, for small-scale studies [23, 25].

### Technical variation

Biological systems are inherently complicated and thus several measurements are typically made in order to establish the distribution of values that represent individual properties. Thus, the convention for six or more experimental measurements (true measures) also applies to phospholipidomics studies. However, technical replicates (repeated measurements of the same sample) in modern NMR are not required. NMR instruments are calibrated through regular QC runs to check shimming, locking etc. At the beginning of a sample run the system is locked to calibrate the chemical shift and shimmed to a specification. 90 degree pulses are calibrated every day and sometimes more frequently to ensure radio frequency (RF) performance and the RF circuits are tuned and matched for each sample. Environmental conditions, such as temperature, are also controlled and faults reported. This fixes the “baseline” so each sample is starting from the same point. Faults with drifting RF would result in very noticeable effects, RF is in megahertz (MHz) we detect Hz so small errors would cause very large changes, this is an example of real time checking. Additionally, each NMR experiment is a stand-alone experiment in which we signal average over >1000 experiments. These factors mean that conventions in biological studies for repeated experimental measurements are required, but repeated measurements of the same sample are not. Details of how samples change with age is described next.

### Sample age/storage

The shelf-life of samples after preparation for data acquisition is of interest as it dictates the size of analytical batches and the timing of data collection. Several aspects of the sample format, such as the relationship between the analytes and the solvent, affect both sample preparation procedures and sample shelf-life. Lipids have several known chemical sensitivities, including to chloroform and bases [13, 39]. We therefore sought to characterize the relationship between sample age and the lipid profile observed in order to determine the optimum range and consequences for the sample outside of this.

**Fig. 3:**
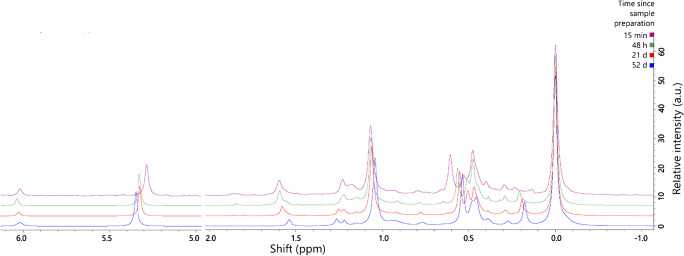
Sample age scan. Samples comprised Lecithin from *Glycine max*, run 15 min, 48 h, 21d and 52d after sample preparation. Samples prepared in the modified CUBO solvent system, 650 μL/sample (13, 22), run and stored at 293 K out of direct sunlight. σ_P_ (324 MHz, CUBO, ppm) 6·1–5·9, *lyso*-phosphatidic acid; 5·4–5·2, phosphatidic acid; 1·65–1·55, lyso-phosphatidylinositol; 1·28–1·20, phosphatidylglycerol; 1·15–0·95, phosphatidylglycerol; 0·85–0·80, sphingomyelin; 0·60–0·57, phosphatidylethanolamine; 0·57–0·55, phosphatidylethanolamine; 0·55–0·51, phosphatidylethanolamine; 0·50–0·48, phosphatidylethanolamine; 0·46–0·43, *lyso*-phosphatidylcholine; 0·30–0·25, unk.; 0·19–0·16 unk.; 0·00, phosphatidylcholine (calibration reference)

The first test was to run the same set of samples shown in Fig. 2 after 21 days to identify any significant changes in lipid profile (ESM Fig. S2). The results of this test showed little gross change, though there is evidence for some minor changes. In order to identify the changes that occur with age, we constructed a time-scan of lecithin (Fig. 3). This comprised four time points, the first of which was at just ~15 min of exposure to the solvent. The results showed a time-dependent change in the chemical shift of PA, PE and PI but no change in integration and was reflected in the ageing of samples of lipids extracted from tissues, such as mouse brain (ESM Fig. S3). One effect evident in this scan was an increase in the size of the LPC signal with respect to PC (referenced to the same integration and shift, 0·00 ppm). This was investigated next.

The increase in abundance of LPC with sample age was consistent with at least one anecdotal observation [17] and led us to the hypothesis that base-mediated hydrolysis of ester bonds occurred, presumably driven by triethylamine. It is useful to test this hypothesis because changes to the lipid profile with age are relevant for interpretation of data and consistent sample handling. The relationship between PC and LPC at 48 h and at 21d over the sample concentration range of 1–32 mg showed that the ratio of LPC to PC was 50–60% higher at 21d than at 48 h across the concentration range tested (ESM Fig. S4). This suggests that hydrolysis of PC increases with sample age but not concentration and is tolerable in the optimum range for S/N of 4–16 mg. This leads us to suggest that in addition to a mass of 4–16 mg for typical mixed samples, data acquisition within one week will provide reliable data without significant hydrolysis.

### Pulse sequence

The CUBO solvent mixture is designed to facilitate sample preparation with a high concentration of lipids in a mono-disperse format and relatively low viscosity, minimizing T1 and T2. T1 is field-dependent, with values ranging from 8·4 s (800 MHz, 700 MHz) [23] to 6·5 s (400 MHz) [22].

For 1D NMR experiments, it is important to acquire the spectrum under quantitative conditions. Specific attention is needed for the decoupling methods. Inverse gated decoupling is needed to avoid NOE transfer. Additionally, sufficient relaxation time (T1) is required to give full recovery. In all our experiments, we used the WALTZ-16 decoupling with a relaxation delay of 5 × T1.

2D ^31^P-^1^H correlation experiments were run using an HSQC method. On cooled probes, good results were achieved with the XY16-CPMG sequence. XY16-CPMG is a low-power composite 180° pulse train that was applied to both ^1^H and ^31^P nuclei during the evolution of long-range couplings as a way to eliminate phase distortions caused by homonuclear proton-proton couplings that evolve at the same time. The indirect ^31^P dimension suffers from low chemical shift dispersion. This problem can be compensated for by running a higher number of points in the indirect dimension (*t*1). The upper limit of the number of points is limited by the relaxation time of ^31^P (T2). We found 2048 points was a good compromise. To reduce acquisition times to reasonable lengths, non-uniform sampling methods can be deployed. The resolution allowed real-world discrimination of ^31^P signals that are separated by as little as 0·025 ppm (5·6 Hz, 324 MHz ^31^P).

### Signal identification: Resonances

A critical step in interpreting metabolomics data is assigning the signals acquired to the appropriate molecular species. In the context of ^31^P NMR for lipidomics, this means assigning resonances to lipid head groups. Previous studies have already analysed milk [21], lecithin [19] and seed extract [13] samples. However, several important tissue types that are desirable for lipidomics studies are not yet described, and it is not clear whether it is possible to align acquisitions taken on different instruments decades apart. This led us to codify the shifts for as many naturally-occurring lipid classes as possible in a range of tissues in one analytical batch on one instrument.

**Fig. 4:**
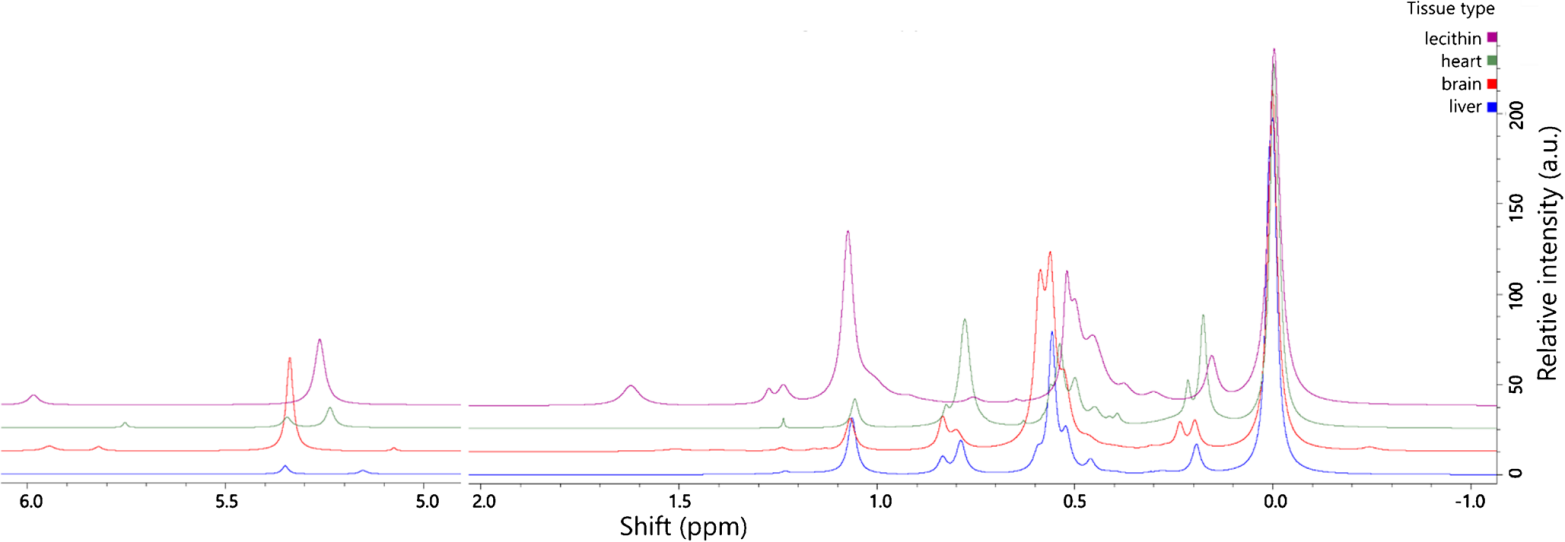
Sample type plot. Samples comprised lipid extracts from either Lecithin extract from *Glycine max* or heart, liver or brain tissues from *Mus musculus*. Samples were prepared in the modified CUBO solvent system, 650 μL/sample (13, 22), and run at 293 K and within 48 h of sample preparation. σ_P_ (324 MHz, CUBO, ppm) 6·1–5·9, *lyso*-phosphatidic acid; 5·4–5·2, phosphatidic acid; 1·65–1·55, lyso-phosphatidylinositol; 1·28–1·20, phosphatidylglycerol; 1·15–0·95, phosphatidylglycerol; 0·85–0·80, sphingomyelin; 0·60–0·57, phosphatidylethanolamine; 0·57–0·55, phosphatidylethanolamine; 0·55–0·51, phosphatidylethanolamine; 0·50–0·48, phosphatidylethanolamine; 0·46–0·43, lyso-phosphatidylcholine; 0·30–0·25, unk.; 0·19–0.16 unk.; 0.00, phosphatidylcholine (calibration reference)

One dimensional ^31^P NMR spectra were collected for lipids extracted from liver, heart and brain (*Mus musculus*) and lecithin (*Glycine max*). Spectra showed that the lecithin contained phospholipids that comprised diglyceride moieties but not ether-glycerides or sphingosines, agreeing with previous reports [40]. The lipids appear in different proportions across samples, clarifying signals associated with mid-sized and minor peaks (Fig. 4). A list of resonances is shown in ESM Table S1. Our approach also represents an advance in assigning signals as it allows us to understand how individual lipids behave in different mixtures as well as by concentration. However, ambiguities remain. Particularly difficult is the busy region 0·45–0·60 ppm in which there are known to be signals for PE-plasmalogen, PE (×2), PS and LPC [13, 18–21, 38]. This led us to use 2D spectra to assign the signals in these samples.

**Fig. 5:**
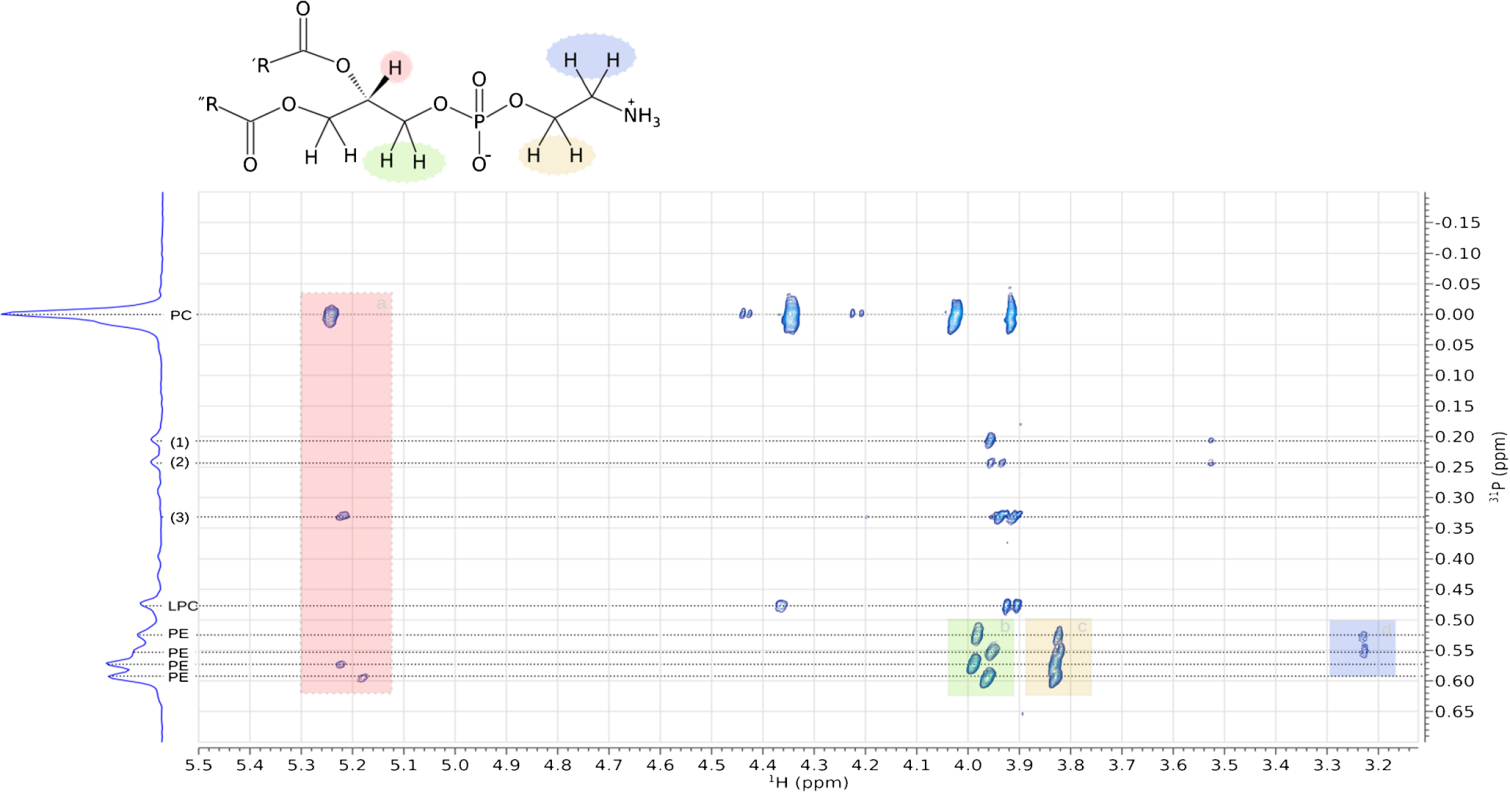
^31^P-HSQC spectrum of lipids extracted from brain of *Mus musculus* showing coupling between P and H nuclei in the phosphate diester, and glyceryl and choline/ethanolamine moieties. Resonances that cannot be reconciled are demarked with number is parentheses (1–3). Resonances that concurred with previous assignments (18–21, 39) are labelled. Inset molecular structure is of the head group of phosphatidylethanolamine with proton environments marked and colour-coded with the spectrum (a-d). σ_H_ (800 MHz, CUBO) ~5·2 ppm (**a**, red), (RO)CH_2_-(RO)C**H**-CH_2_-O-P; ~4·4 ppm, CH_2_-O-P(O)(O^−^)-O-CH_2_C**H**_**2**_N(CH_3_)_3_ (PC/LPC); ~4·0 ppm (**b**, green), (RO)CH_2_-(RO)CH-C**H**_**2**_-O-P; ~3·9 ppm, CH_2_-O-P(O)(O-)-O-C**H**_**2**_CH_2_N(CH_3_)_3_ (PC/LPC); ~3·85 ppm (**c**, beige), CH_2_-O-P(O)(O-)-O-C**H**_**2**_CH_2_NH_3_^+^; ~3·2 ppm (**d**, blue) CH_2_-O-P(O)(O^−^)-O-CH_2_C**H**_**2**_NH_3_^+^ (PE). LPC, *lyso*-phosphatidylcholine; PC, phosphatidylcholine; PE, phosphatidylethanolamine

**Fig. 6:**
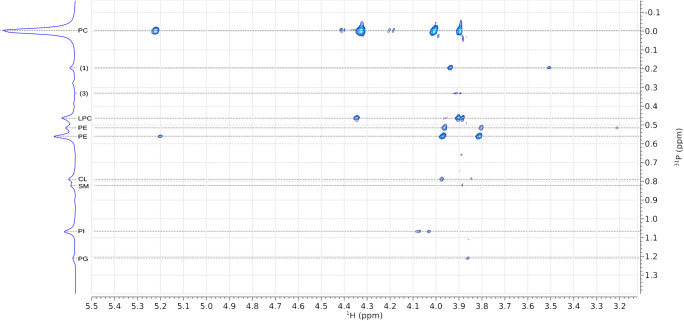
^31^P-HSQC spectrum of lipids extracted from liver of *Mus musculus* showing coupling between P and H nuclei in the phosphate diester, and glyceryl and choline/ethanolamine moieties. Resonances that cannot be reconciled are marked with number is parentheses (1, 3; numbering is consistent with Fig. 5). Resonances that concurred with previous assignments (18–21, 39) are labelled. CL, cardiolipin; LPC, lyso-phosphatidylcholine; PC, phosphatidylcholine; PE, phosphatidylethanolamine; PG, phosphatidylglycerol; PI, phosphatidylinositol; SM, sphingomyelin

**Fig. 7:**
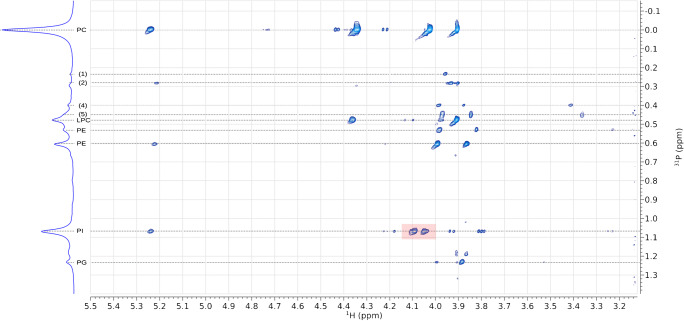
^31^P-HSQC spectrum of lipids extracted from *Glycine max* (soya lecithin) showing coupling between P and H nuclei in the phosphate diester, and glyceryl and choline/ethanolamine moieties**.** Resonances that cannot be reconciled are marked with number is parentheses (1, 2, 4, 5; numbering consistent with Figs. 5 and 6). Resonances that concurred with previous assignments (18–21, 39) are labelled. The red box shows the resonances for the protons nearest the P atom in PI, (RO)CH_2_-(RO)CH-C**H**_**2**_-O-P(O)(O-)-O-C**H**R_2_. CL, cardiolipin; LPC, *lyso*-phosphatidylcholine; PC, phosphatidylcholine; PE, phosphatidylethanolamine; PG, phosphatidylglycerol; PI, phosphatidylinositol; SM, sphingomyelin

We used 2D NMR (^1^H/^31^P HSQC) to determine P-H couplings and assign resonances and thus identify head groups. Three biological samples, mouse brain (Fig. 5), mouse liver (Fig. 6) and soya lecithin (Fig. 7) were used. HSQC spectra of the brain extract showed four strong correlations of the phosphorus atom associated with phosphatidylcholine with proton environments and four much weaker ones that form two pairs (*σ*_*P*_*,* 0·00 ppm, Fig. 5). Using the known molecular structure of this phospholipid (inset, Fig. 5), the stronger signals were assigned to the two methylenes of the choline head group, the *sn-*3 methylene and *sn-*2 methine of the glyceryl moiety. A 2D spectrum of mouse plasma that consists chiefly of LPC and PC informed this assignment. These two lipids have known referenced 1D shifts of ~0·48 and 0·00 ppm, respectively (ESM Fig. S5). LPC does not have a resonance at *σ*_*H*_ 5·2 ppm and the resonance at 4·0 ppm appears slightly upfield at 3·9 ppm. The signal at *σ*_*H*_ 5·2 ppm is characteristic of the methine proton in a glyceryl moiety of a glyceride [41], and thus its disappearance with a slight shift upfield of the neighbouring signal for the methylene associated with the *sn-3* position are consistent with a fatty acid residue being removed from the *sn*-2 OH in the glyceryl moiety.

The resonances in the 0·60–0·45 ppm region include important phospholipids such as PE and PS and for most mammalian and other samples it is important to be able to assign these unambiguously. Mouse brain and liver and soya lecithin samples are ideal for this as they comprise the appropriate lipids in sufficient abundance to show clear resonances but those abundances also differ from one another. Magnifications of this region of the 2D brain spectrum showed two pairs of signals, one pair coupling with additional signals at 3·2 ppm in the proton spectrum (Fig. 5). This is consistent with previous reports that suggested and even showed that interaction of the PE head group with the solvent system results in several signals [21–23, 38]. The signals observed in this 2D spectrum (Fig. 5) deepen our understanding of this phenomenon as they provide a possible explanation for the presence of several signals through different conformers. For example, if the primary amine in PE is protonated, it is favourable for it to form attractive electrostatic interactions with the anionic phosphate group. However, if it is deprotonated, the lone pairs are exposed and there will be a repulsion between the amine and phosphate. The exposed primary amine and phosphate may therefore interact with electropositive systems such as triethylammonium or guanidinium ions, or metal ions such as that of sodium. This complicated set of possibilities led us to investigate the phenomenon of adducts in these samples, i.e. electrostatic interactions between the lipids and the solvent system.

### Signal identification: Adducts

An advantage of ^31^P NMR over mass spectrometry is that analytes do not need to be ionized to be detected. However, ionic compounds such as those containing a phosphate interact with local ions through electrostatic attraction. This changes how the ^31^P nucleus is deshielded and thus alters the chemical shift its resonance(s) adopt. The interaction of analytes with the solvent system is understood to be the reason that more than one resonance is observed for lipids such as PE. To simplify the job of assigning the signals from ^31^P NMR data, an extraction that minimizes the range of adducts is suggested, e.g. favouring triethylammonium [13, 15]. The latter adduct is entirely compatible with the CUBO solvent system and generally offers better solvation in organic solvents. However, some sodiated, potassiated or ammoniated analytes may also be present depending upon sample handling. The presence of guanidinium ions also offers scope for electrostatic interaction.

**Fig. 8:**
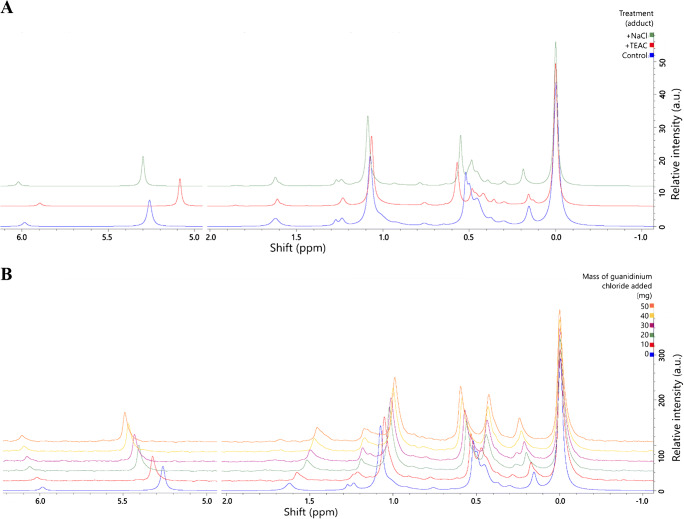
Sample adduct plot. Panel A, samples comprised lipid extracts from *Glycine max* that were made as normal or exposed to NaCl (10 mg) or triethylammonium chloride (10 mg) after being made; B, samples comprised lipid extracts from *Glycine max* that were made as normal or exposed to guanidinium chloride (10–50 mg) was added to aliquots of a sample stock (10 mg lecithin, 650 μL solvent per sample). Samples were prepared in the modified CUBO solvent system with a final volume of 650 μL/sample (13, 22), and run at 293 K and within 72 h of sample preparation. σ_P_ (324 MHz, CUBO, ppm) 6·1–5·9, *lyso*-phosphatidic acid; 5·4–5·2, phosphatidic acid; 1·65–1·55, *lyso*-phosphatidylinositol; 1·28–1·20, phosphatidylglycerol; 1·15–0·95, phosphatidylinositol; 0·85–0·80, sphingomyelin; 0·60–0·57, phosphatidylethanolamine; 0·57–0·55, phosphatidylethanolamine; 0·55–0·51, phosphatidylethanolamine; 0·50–0·48, phosphatidylethanolamine; 0·46–0·43, *lyso*-phosphatidylcholine; 0·30–0·25, unk.; 0·19–0·16 unk.; 0·00, phosphatidylcholine (calibration reference)

We tested the hypothesis that some signals could be ascribed to sodiated lipid adducts by adding sodium chloride (10 mg) to the sample solution preparation tube, before decanting into the glass sample tube. The same test for triethylammonium and guanidinium adducts was done by adding triethylammonium chloride (1 sample, +10 mg) and a range of masses of guanidinium chloride (5 samples, 10–50 mg/sample) respectively. The results of this test showed that the presence of signals for LPA, PA, PI and PE all differ relative to PC for both triethylammonium and sodium ions (Fig. 8A). A scan of guanidinium concentration showed that this adduct also modulated the shift of several lipids, but not always in the same direction as either triethylammonium or sodium (Fig. 8B). This showed that interaction with ions is essential, but also suggests that the interaction with the solvent and local ions is not straightforward. It underscores the need for consistent handling within and between analytical batches. Our recommendations for minimizing variation between samples are therefore for extractions to be carried out in parallel using the same procedure with solvents and handling procedures carried out to minimize the addition of adducts other than TEAC and guanidinium. This will also facilitate assignment of resonances.

### Quantification: Signal deconvolution

The solvent system used in the present study offers the best separation and resolution of signals of the three types of solvent system currently reported (CUBO, chloroform/methanol, aqueous detergent), however, some signals remain relatively close to others and thus a clearer approach is needed before the area of the resonance can be measured and thus the relative molar concentration determined. As discussed above, resonances for PE-plas, PS, LPC and two for PE appear 0·60–0·45 ppm. A useful method for quantification (allied to signal identification, *vide supra*) is to use the deconvolute (dcon) function in Bruker’s TopSpin software. This assigns a known lineshape to peak-picked resonances, which facilitates quantification for all signals in the spectrum. An appropriate lineshape (Gaussian, Lorenzian or a compromise between them) allows resonances to be integrated and thus the abundance of that head group to be determined. A list of integrations can then be created (%mol of the total) and exported to a spreadsheet workbook.

## Conclusion

In this paper we have described a pipeline for using ^31^P NMR for lipidomics. This makes the technique more accessible for hypothesis-driven research and new users. Several existing questions about the use of this technique for profiling the lipid composition of biological samples have been answered, including which resonances represent what lipids, how concentration and ageing affect samples and how much material is required for a useful spectrum. This provides all of the tools necessary for both new and experienced NMR spectroscopists to use this technique to answer scientific questions that require lipidomics data. It also dovetails neatly with lipidomics data collected by mass spectrometry for dual spectroscopy.

## Supplementary Information

ESM 1 (DOCX 261 kb)

## Data Availability

The raw data for the spectra used in this study are publicly available from not after 16/05/2022 from EBI’s BioStudies platform, accession number S-BSST651.
