# Supplementary material for: A pipeline for making 31P NMR accessible for small- and large-scale lipidomics studies
Source: Anal Bioanal Chem. 2021 Jul 12;413(19):4763–73. doi: 10.1007/s00216-021-03430-4 (PMC8318958; doi:10.1007/s00216-021-03430-4)
Supplement: Supplementary file 1 — (DOCX 261 kb) [file 216_2021_3430_MOESM1_ESM.docx]

**Supplementary Information**

Supplementary Figures

**

***Fig. S1. Signal-to-noise plots of head group resonances from phosphorus atoms in spectra of samples of lecithin from* Glycine max.** *Concentrations 1-32 mg. Data acquired at 48h after sample preparation. Samples were prepared in the modified CUBO solvent system [*[*13*](#_ENREF_13)*,*[*22*](#_ENREF_22)*] and both run and stored at 293K. The correlations of all head groups across the full range were r ≥ 0·99 except for PS (r = 0·97). For the range 4-16, all lipid head groups had correlations of r ≥ 0·99. CL, cardiolipin; PC, phosphatidylcholine; PE, phosphatidylethanolamine (1 and 2) ; PG, phosphatidylglycerol; PI, phosphatidylinositol.*

*
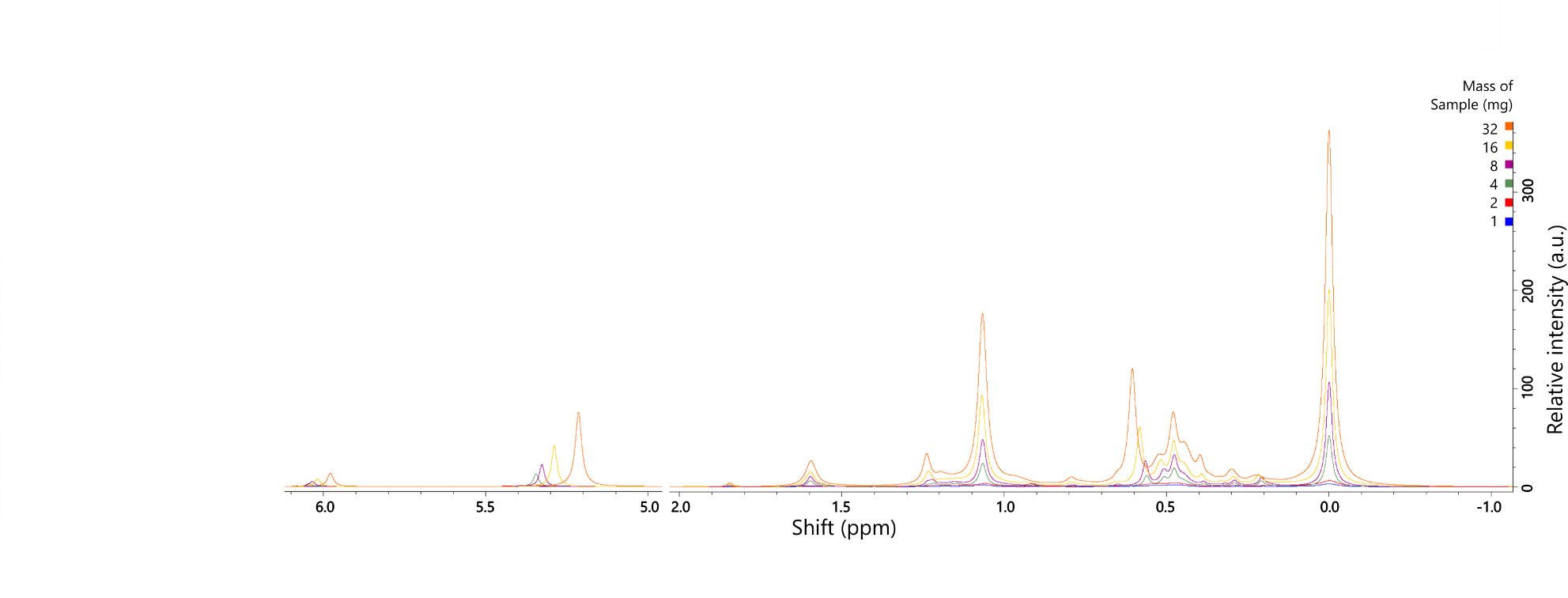
*

***Fig. S2. Sample concentration scan.*** *Samples comprised Lecithin from* Glycine max*, data acquired 21d after sample preparation. Samples prepared in the modified CUBO solvent system, 650 µL/sample [*[*13*](#_ENREF_13)*,*[*22*](#_ENREF_22)*], acquired at 293K. σ_P_ (324 MHz, CUBO, ppm) 6.1-5.9,* lyso-*phosphatidic acid; 5.4-5.2, phosphatidic acid; 1.65-1.55,* lyso-*phosphatidylinositol; 1.28-1.20, phosphatidylglycerol; 1.15-0.95, phosphatidylinositol; 0.85-0.80, sphingomyelin; 0.60-0.57, phosphatidylethanolamine; 0.57-0.55, phosphatidylethanolamine; 0.55-0.51, phosphatidylethanolamine; 0.50-0.48, phosphatidylethanolamine; 0.46-0.43,* lyso-*phosphatidylcholine; 0.30-0.25, unk.; 0.19-0.16 unk.; 0.00, phosphatidylcholine (calibration reference).*

*
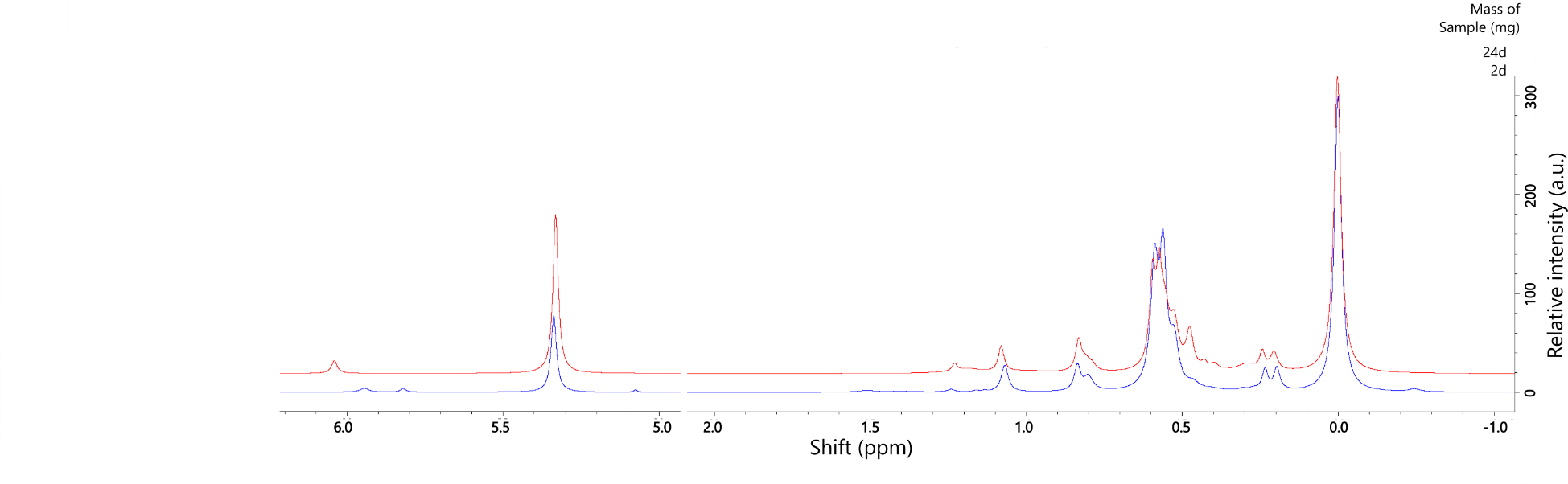
*

***Fig. S3. Sample age scan.*** *Samples comprised lipid extract from brain from* Mus musculus*, run 48h and 24d after sample preparation. Samples prepared in the modified CUBO solvent system, 650 µL/sample [*[*13*](#_ENREF_13)*,*[*22*](#_ENREF_22)*], run and stored at 293K out of direct sunlight. σ_P_ (324 MHz, CUBO, ppm) 6.1-5.9,* lyso-*phosphatidic acid; 5.4-5.2, phosphatidic acid; 1.65-1.55,* lyso-*phosphatidylinositol; 1.28-1.20, phosphatidylglycerol; 1.15-0.95, phosphatidylinositol; 0.85-0.80, sphingomyelin; 0.60-0.57, phosphatidylethanolamine; 0.57-0.55, phosphatidylethanolamine; 0.55-0.51, phosphatidylethanolamine; 0.50-0.48, phosphatidylethanolamine; 0.46-0.43,* lyso-*phosphatidylcholine; 0.30-0.25, unk.; 0.19-0.16 unk.; 0.00, phosphatidylcholine (calibration reference).*

**

***Fig. S4. Plots of the ratio of the integration of LPC and PC resonances as samples age.*** *Samples comprised Lecithin from* Glycine max*, run 48h after sample preparation. Samples prepared in the modified CUBO solvent system, 600 µL/sample [*[*13*](#_ENREF_13)*,*[*22*](#_ENREF_22)*], run and stored at 293K out of direct sunlight. LPC,* lyso-*phosphatidylcholine; PC, phosphatidylcholine.*

*
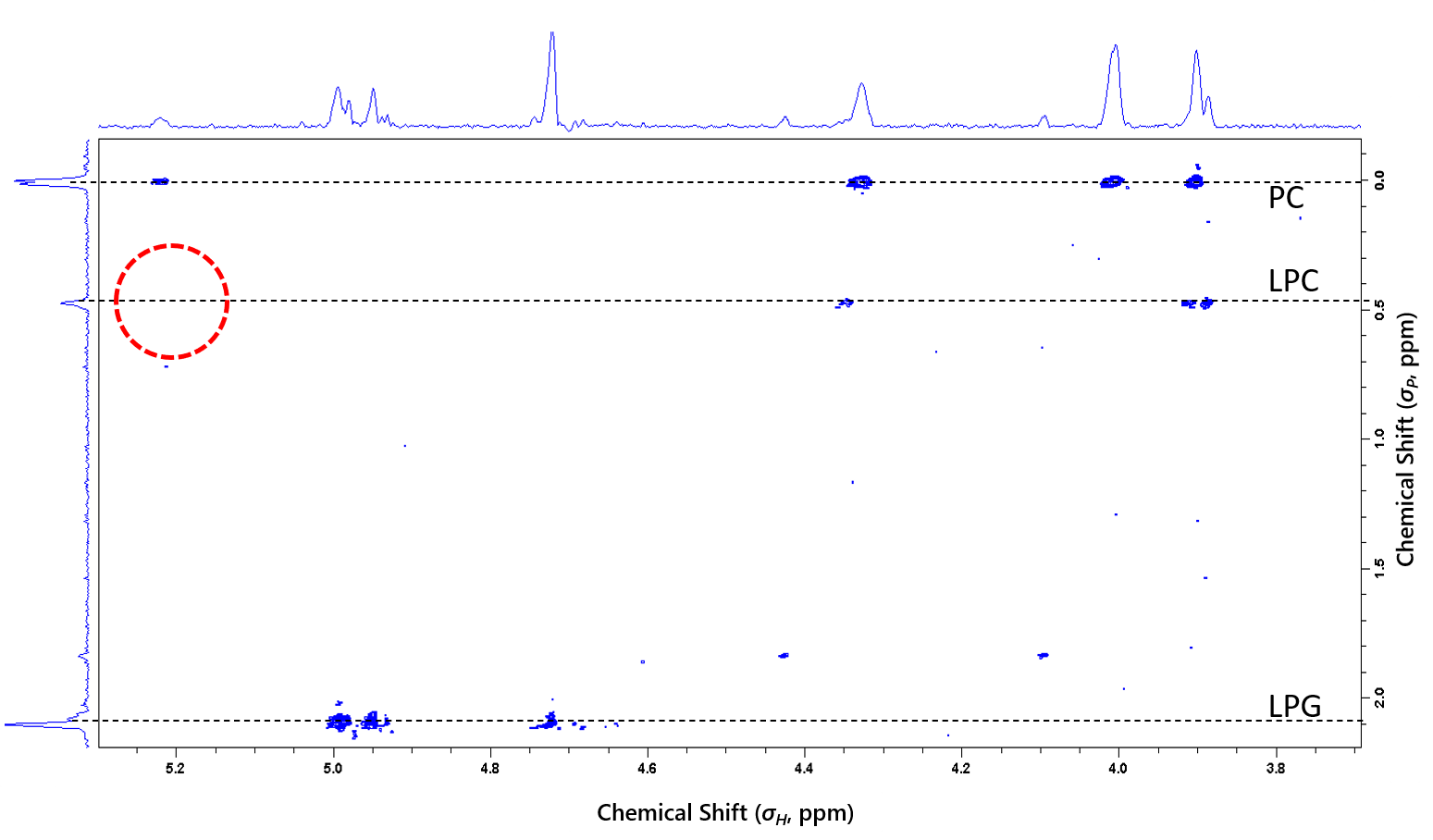
*

***Fig. S5.*** *^31^P-HSQC spectrum of lipids extracted from plasma of* Mus musculus *showing glyceryl and choline resonances from phosphatidylcholine,* lyso-*phosphatidylcholine and* lyso-*phosphatidylglycerol. Dashed red line indicates where the C*H *resonance that appears for PC but does not in LPC. LPC,* lyso-*phosphatidylcholine; LPG,* lyso-*phosphatidylglycerol; PC, phosphatidylcholine.*

| Shift (ppm) | Assignment | Notes |
| --- | --- | --- |
| 0.00 | Phosphatidylcholine (PC) | Spectra calibrated to PC at 0·00 ppm;  Sometimes split |
| 0·06 | Unknown | Previously assigned to plasmalogen-PC. |
| 0·16 | Unknown (1) |  |
| 0·31 | Unknown (2) |  |
| 0·38 | Unknown (4) |  |
| 0·46 | Unknown (5) |  |
| 0·50 | *lyso-*Phosphatidylcholine (*lyso-*PC, LPC) |  |
| 0·52 | Phosphatidylethanolamine (PE) | Shift dependent upon sample age and concentration, and guanidine concentration |
| 0·54 | Phosphatidylethanolamine (PE) | Shift dependent upon sample age and concentration, and guanidine concentration |
| 0·77 | Cardiolipin (CL) |  |
| 0·83* | Sphingomyelin (SM) | Frequently split |
| 1·08 | Phosphatidylinositol (PI) | Shift dependent upon guanidine concentration and sample age but not sample concentration |
| 1·24  1·27 | Phosphatidylglycerol (PG) | Usually split (uneven) |
| 1·49 | *lyso-*Phosphatidylinositol (*lyso-*PI, LPI) |  |
| 1·62 | *lyso-*Phosphatidylglycerol (*lyso-*PG, LPG) | Typically appears with a resonance at 1·82 ppm, assigned to 1-*O*- isomer |
| 5·26 | Phosphatidic acid (PA) | Shift dependent upon sample age and concentration, and guanidine concentration |
| 5·99 | *lyso-*Phosphatidic acid (*lyso-*PA, LPA) | Shift dependent upon sample age and concentration, and guanidine concentration |

***Table S1.*** *List of chemical shifts of resonances of phosphorus environments in phospholipids found in lecithin from Glycine max. *Found in mammalian samples. These interpretations are based on literature precedents [*[*18-21*](#_ENREF_18)*,*[*39*](#_ENREF_34)*] and 2D data collected in the present study (Figs. 5-7, S5).*
